# Leader–Follower Approach for Non-Holonomic Mobile Robots Based on Extended Kalman Filter Sensor Data Fusion and Extended On-Board Camera Perception Controlled with Behavior Tree

**DOI:** 10.3390/s23218886

**Published:** 2023-11-01

**Authors:** Arpit Joon, Wojciech Kowalczyk

**Affiliations:** Institute of Automatic Control and Robotics, Poznan University of Technology, Piotrowo 3A, 60-965 Poznan, Poland; wojciech.kowalczyk@put.poznan.pl

**Keywords:** sensor data fusion, fiducial marker, extended kalman filter, mobile robots, leader–follower control, ROS, landmarks, behavior tree

## Abstract

This paper presents a leader–follower mobile robot control approach using onboard sensors. The follower robot is equipped with an Intel RealSense camera mounted on a rotating platform. Camera observations and ArUco markers are used to localize the robots to each other and relative to the workspace. The rotating platform allows the expansion of the perception range. As a result, the robot can use observations that are not within the camera’s field of view at the same time in the localization process. The decision-making process associated with the control of camera rotation is implemented using behavior trees. In addition, measurements from encoders and IMUs are used to improve the quality of localization. Data fusion is performed using the EKF filter and allows the user to determine the robot’s poses. A 3D-printed cuboidal tower is added to the leader robot with four ArUco markers located on its sides. Fiducial landmarks are placed on vertical surfaces in the workspace to improve the localization process. The experiments were performed to verify the effectiveness of the presented control algorithm. The robot operating system (ROS) was installed on both robots.

## 1. Introduction

Different types of robots [1,2] are created by researchers according to specific tasks and applications. They have also developed different types of controllers for collision avoidance [3] and also for leader–follower configurations [4]. Due to their simplicity and ease of use, fiducial ArUco markers are widely used. Researchers have proposed different types of control algorithms [5,6] with different features depending on their applications.

The pose calculation from the ArUco marker using the Kalman Filter is well described in the paper [7]. The paper [8] explains a tutorial on behavior trees in video games and the book [9] gives an introduction to the behavior tree. The examples of controls for the formation of robots are described in the papers [10,11]. The paper [12] gives an overview of the introduction of the landmarks with the Extended Kalman Filter.

Control based on only onboard sensors is more challenging but results in a robot’s operating area that is not limited by the coverage of the external measurement system. The novelty of the paper is an approach of controllers for leader–follower robots including the independent control of the camera’s field of view. While the rotating platform does not cover the full angle (its range is −150 to 150 degrees), it allows observation of a robot and landmarks located on the sides or to some extent behind. This is achieved by using only one camera. The main task of the behavior tree is to switch between observing the leader and static fiducial markers placed on the wall. This provides correction of positions obtained based on intrinsic sensors, which can drift, using features available in the environment. In addition, the decision to switch between observed objects using a behavior tree will make it possible in the future to easily shape the process of perception of the environment depending on the conditions of the task (for example, the speed of robots and other objects, the quality of perception due to the distances and angles of observation of markers or lighting conditions).

An additional benefit of using a behavior tree is the simplified management of task initiation. In the case of tasks carried out by more than one robot, it is necessary to spread information about the readiness of key sub-systems to work. This problem can be solved in many ways and is usually overlooked in the literature. It has been faced by every team implementing a multi-robot system. This paper proposes a neat solution to this problem based on the behavior tree approach.

To the best of the authors’ knowledge, there is no use of a behavior tree in the literature to expand the robot’s perception range. In particular, the use of this approach in a multi-robot system has never been proposed. According to the authors, the proposed combination of hardware and software allows for a significantly reduced cost of the perceptual system because there is no need to use many cameras.

The authors emphasize that the practical usefulness of this approach is not obvious, because the rotation of the camera reduces the quality of the recorded image, and as a result, makes it difficult to track the marker. The research presented in the article shows that under standard laboratory conditions, the camera on a rotating platform does work and can be used in this kind of task. The ground points which explain the difference between already published papers and work represented by authors in this paper are as follows:

(a) A decentralized vision-based system for markers is represented in the paper [13]. In the same paper, each robot has a truncated regular octagon and a fixed camera, whereas in this paper, the camera is movable and independent of the movement of the mobile platform.
(b) The paper [14] is the extension of the research work presented in paper [13]. A dual Unscented Kalman Filter is used to estimate the leader’s velocities from fiducial markers. But the work presented in this paper has a centralized architecture and only simulation results are given.
(c) In the paper [15], the ArUco markers are placed on the top of the robots and the camera is placed on the stand observing the robot vertically. The disadvantage of the method presented in this paper is that the vertical camera should cover all the environments with the robots. Our algorithm presented in this paper covers this gap and such a solution limits the working area only to that covered by the measurement system.
(d) The paper [16] represents a hitchhiking robot approach for leader–follower robots. The author used a QR marker, and the mapping and computation are conducted by the driver robot. But a completely different control algorithm from this paper is used with a fixed camera.

The paper [17] used the ArUco markers on the top of the robots which presents a protocol for a network of multiple robots. The nonholonomic robots in the paper are asymptotically stabilized. The paper [18] represents an algorithm for intermittent vision measurements and odometry with noise. The follower robot generates the trajectory of the leader robot with Bayesian trajectory estimation, and the ArUco marker was on the leader robot.

The paper [19] represents a fusion system with the combination of technologies INS, EKF, IMU, GNSS, and information from ArUco markers. The paper [20] represents the follower robot recognizing the ArUco marker on the clothes worn by the factory worker. The Mecanum platform is used and the human gait error model is also presented by the authors. The paper [21] also presents an algorithm with ArUco markers placed on the assets of a truncated rhombi cuboctahedron on the drone. The GPS-denied environment for Unmanned Aerial Systems (UASs) is discussed in the paper.

This paper is the extension of the conference paper [22] with landmark fusion in EKF and a behavior tree controlling the robots. A leader–follower mobile robot control approach using onboard sensors and fiducial markers is shown in this paper. A 3D-printed cuboidal tower is mounted on the leader robot with four ArUco markers located on its sides. The follower robot is equipped with an Intel RealSense camera mounted on a rotating platform. An Intel RealSense Depth sensor D435 is installed on the follower robot. The depth sensor has an ideal range from 0.3 m to 3 m which works in indoor as well as in outdoor environments. Both the leader and follower robots are equipped with MPU9255 IMU sensors which deliver nine pieces of information, a three-axis gyroscope, a three-axis accelerometer, and a three-axis compass/magnetometer. A Dynamixel 12-A servo motor is used in a one-degree-of-freedom rotating platform. The servo motor has a resolution of 0.29 degrees per pulse with 0 to 300 degrees of endless turn. Both of the robots also have an NUC Intel single-board PC which has inbuilt Wi-Fi modules for connectivity. Camera observations and ArUco markers are used to localize the robots to each other and relative to the landmarks located in the workspace. A proportional–integrative–derivative (PID) controller rotates the camera platform independently from the mobile platform movement of the follower robot. The follower robot detects the ArUco markers on the leader robot and calculates its pose. The data from the wheel encoders and the IMU sensors are fused by EKF to calculate the global pose of the robots in the environment. The environment has artificial markers (ArUco markers) on vertical surfaces. The EKF of the follower robot also takes the data from the landmarks to update its pose.

**Figure 1:**
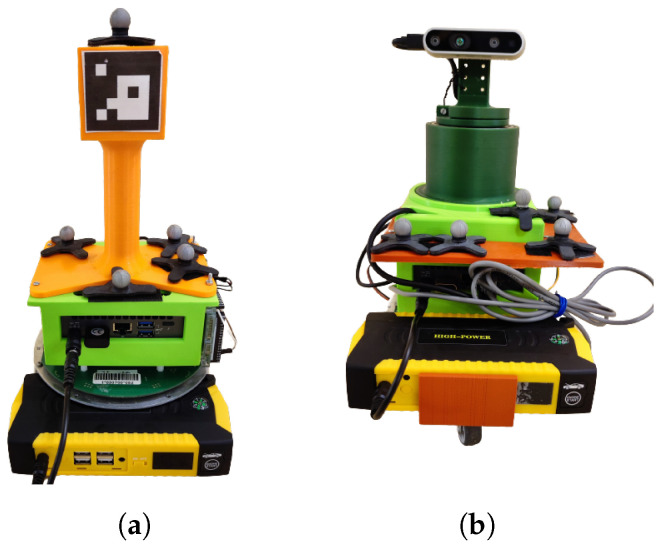
(**a**) Leader robot. (**b**) Follower robot.

The Robot Operating System (ROS) is installed on both robots. The experiments are performed to verify the effectiveness of the control algorithm presented in the paper. The leader robot is shown in Figure 1a and the follower robot is shown in Figure 1b. The behavior tree is explained in Section 2, ArUco marker detection in Section 3, the Extended Kalman Filter and its matrices specific to the sensors used in Section 4, and the control algorithm in Section 5. The experimental methodology is given in Section 6 and the experimental results in Section 7. In the last section, concluding remarks are given.

## 2. Behavior Tree

In general, behavior trees are used in video games, computer science, control systems, and robotics and is defined as the mathematical model to execute a plan.

**Figure 2:**
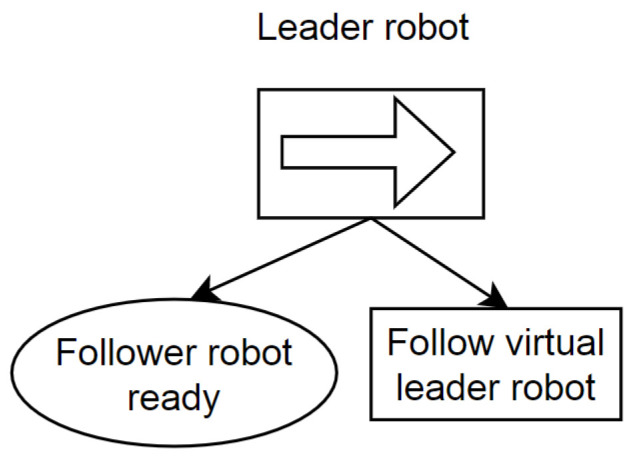
Behavior tree of leader robot.

**Figure 3:**
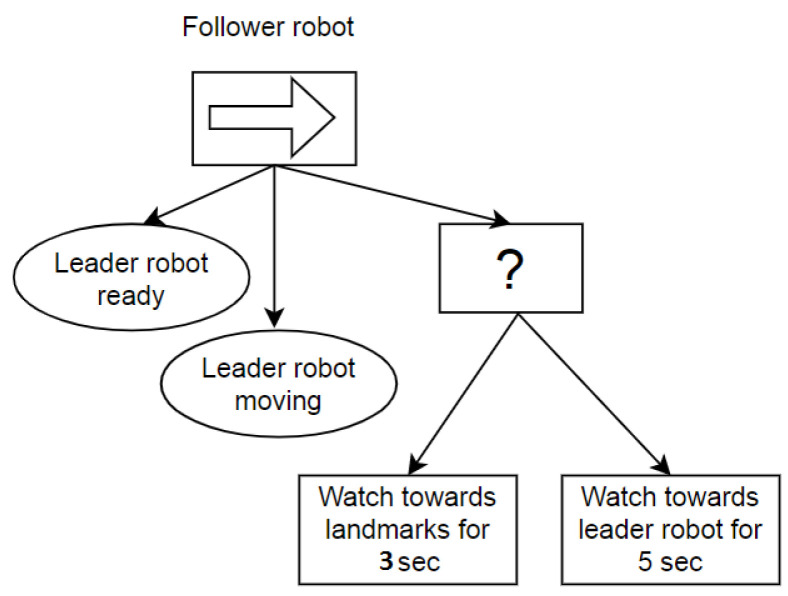
Behavior tree of follower robot.

The plan execution of the leader and follower robot is controlled by the two separate behavior trees implemented in on-board systems. The behavior tree is used as it is effective and easy to modify and extend the tasks in the same trees. The behavior trees only execute the node that is in the current state which makes them effective while executing. Another feature is that behavior trees are easily debuggable, as they are visual representations of the sequence of tasks. The C++ library [23] is used to build and execute the behavior tree in ROS. At the heart of a behavior tree is the root node, which acts as the starting point. From here, the tick signal begins its journey through the tree’s nodes. The initiation of a behavior tree’s operation commences at the root. This root node emits periodic “ticks” to its child nodes. As a node within the behavior tree is permitted to execute, it conveys a status update back to its parent node. If the node’s task is still in progress, it communicates a “running” status, if the objective is accomplished, it communicates “success”, and if the task encounters an obstacle or failure, it relays “failure” to the parent node. The representation of the behavior tree for the leader robot is presented in Figure 2 and for the follower robot in Figure 3. The behavior tree of the leader robot has the condition “Follower robot ready”; only when the follower robot is connected to the ROS master and sends the signal that it is ready to move will this condition relay a “success” status to the upper-level sequence node. After success, the sequence node will execute the task “Follow virtual robot”, in which the leader robot will start moving and follow the trajectory of the virtual robot.

The behavior tree of the follower robot checks the two conditions “Leader robot ready” and “Leader robot moving”. The sequence node will obtain two successes when the leader robot is connected to the ROS master and moving with some velocities. After success from two conditions, the sequence node will execute a fallback node. The fallback node will rotate the camera platform of the follower robot towards the landmarks on walls for 3 s and then rotate the camera platform towards the leader robot for 5 s. The execution time of the platform rotation is negligible from the point of view of the task.

## 3. ArUco Marker

ArUco markers are fiducial markers created by S. Garrido-Jurado [24]. The different IDs and sizes of ArUco markers can be created from the online generator website [25]. The distances and the angles between the ArUco marker and the camera axes can be extracted by using the OpenCV library [26].

Section 3.1 explains the placements of ArUco markers on the leader robot. The data extraction after detection of the ArUco marker is explained in Section 3.3. The calculation of the leader pose expressed in the global coordinate frame is explained in Section 3.4.

### 3.1. ArUco Marker Placement on Leader Robot

**Figure 4:**
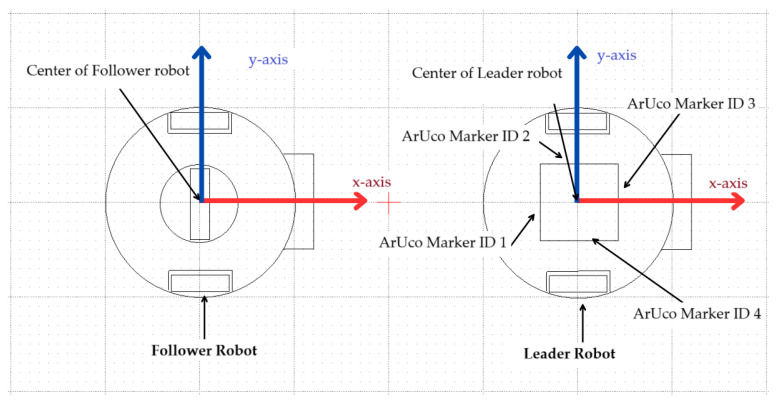
Top view of the leader and follower robots.

The center of the cuboidal tower coincides with the center of the leader robot. The ArUco markers from IDs 1 to 4 of size 5 cm × 5 cm are pasted on the faces of the cuboidal tower (Figure 1a). Different IDs are required to detect the side of the leader robot which has a direct influence on the angle between the follower and leader robot. Figure 4 shows the top view of the leader and follower robot, the same figure also shows the ArUco marker placement on the leader robot.

### 3.2. Artificial Landmarks

ArUco markers of size 10 cm × 10 cm from IDs 100 to 104 are pasted on the vertical surface in the environment. The distance between the landmarks is measured in the environment and the same distances are added according to respective IDs to give global positions in the environment.

### 3.3. Pose Estimation from ArUco Markers

A calibrated camera gives the precise information from the markers, distortion coefficients (*D*), and camera matrix (*K*) of the same camera as follows:

(1) $$D=\left[\begin{array}{ccccc} k1 & k2 & c1 & c2 & k3 \end{array}\right],$$

(2) $$\begin{array}{c} K=\left[\begin{array}{ccc} f_{x} & 0 & c_{x}\\ 0 & f_{y} & c_{y}\\ 0 & 0 & 1 \end{array}\right]. \end{array}$$

Intel’s ROS library [27] helps to connect the Intel RealSense sensor to the ROS environment. The factory calibration information, such as D and K values, is published through the ROS topic “/camera/color/camera_info’’.

A total of two cases are possible when the follower robot observes the leader robot in the camera frame. In case 1 (Section 3.3.1), the follower robot observes only one ArUco marker in the camera frame, or in case 2 (Section 3.3.2), the follower robot observes two ArUco markers at the same time. The translation and the rotation of ArUco markers are conducted to calculate the center of the leader robot.

#### 3.3.1. Case 1—When One Marker Detected

When one ArUco marker on the leader robot comes into the camera frame of the follower robot, ID information and the translation (${\vec{t}}_{vec}$) and rotation (${\vec{r}}_{vec}$) vectors are extracted by the ArUco library [26]. The camera rotation matrix relative to the tag (ArUco marker) is calculated by Rodrigues’ formula as follows:

(3) $$R_{CT}=Rodrigues\left({\vec{r}}_{vec}\right)\;,$$

The transposition of Equation (3) gives the rotation matrix of the tag relative to the camera ($R_{TC}$):

(4) $$R_{TC}=R_{CT}^{T}\;,$$

**Figure 5:**
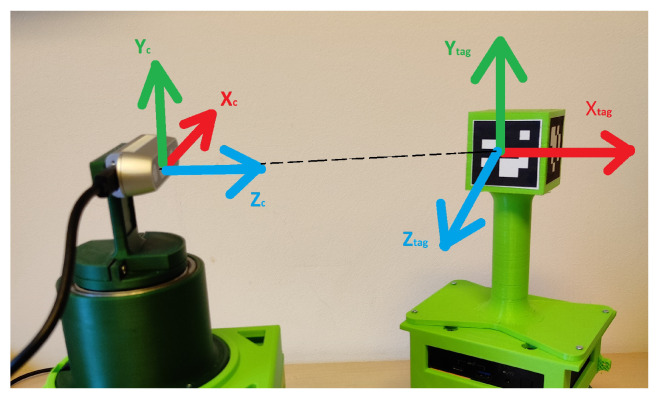
Representation of the axis between the ArUco marker and the camera.

The Euler angles ($\phi_{R}$, $\theta_{R}$, $\psi_{R}$) from the tag to the camera are calculated from the rotation matrix $R_{TC}$. The Z–Y–X rotation conventions are used for Euler angles. The axis representation between the camera and ArUco marker is shown in Figure 5. The leader–follower controller shown in this paper requires the geometric center of the leader and follower robots. The ArUco markers have some offset distance from the center on the leader robot. The translation is conducted to move the marker exactly to the center of the leader robot. The algorithm proposed in this paper to calculate the leader robot’s pose requires that the XY plane of the ArUco marker be parallel to the camera’s XY plane. The first rotation is conducted from the ArUco markers and then the translations. When a rotation matrix $R_{y}\left(\theta_{R}\right)$ is multiplied by the rotation matrix $R_{TC}$, it gives a new matrix $\overset{´}{R_{TC}}$ which is rotated along the y-axis of the marker.

(5) $$R_{y}\left(\theta_{R}\right)=\left[\begin{array}{ccc} \cos\theta_{R} & 0 & \sin\theta_{R}\\ 0 & 1 & 0\\ -\sin\theta_{R} & 0 & \cos\theta_{R} \end{array}\right]\;,$$

(6) $$\overset{´}{R_{TC}}=R_{y}\left(\theta_{R}\right)\cdot R_{TC}\;,$$

To virtually displace the marker from the cuboidal wall to the center of the leader robot which is also the center of the cuboidal tower, a translation should be added in Z-axis. The cuboidal tower measures 0.06 m on each side so 0.03 m is added into the (${\vec{t}}_{vec}$) vector of the marker. The new vector (${\vec{\overset{´}{t}}}_{vec}$) of the marker is as follows:

(7) $${\vec{\overset{´}{t}}}_{vec}={\vec{t}}_{vec}+{\left[\begin{array}{ccc} d_{x} & d_{y} & d_{z} \end{array}]\right.}^{T},$$

where $d_{x}=0$, $d_{y}=0$ and $d_{z}=0.03$.

The translation matrix (${\vec{t}}_{cam}^{\;tag}$) to the camera coordinate is calculated from $\overset{´}{R_{TC}}$ from Equation (6) and ${\vec{\overset{´}{t}}}_{vec}$.

(8) $${\vec{t}}_{cam}^{\;tag}=-\overset{´}{R_{TC}}\cdot {\vec{\overset{´}{t}}}_{vec}\;,$$

The transpose representation of the translation matrix ${\vec{t}}_{cam}^{\;tag\;T}$ is as follows:

(9) $${\vec{t}}_{cam}^{\;tag\;T}=\left[\begin{array}{ccc} x_{marker} & y_{marker} & z_{marker} \end{array}\right]\;.$$

**Figure 6:**
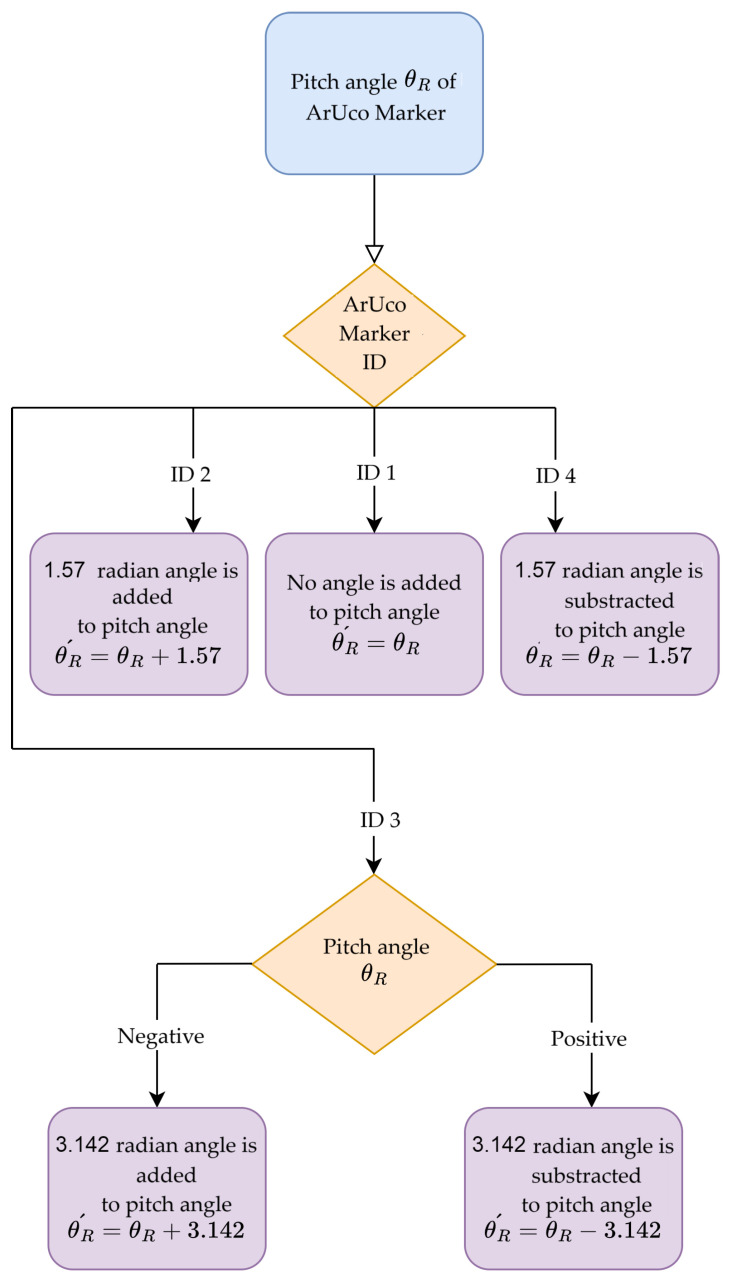
Flow chart of pitch angle $\theta_{R}$ modification of ArUco marker.

$z_{marker}$ and $x_{marker}$ from the translation vector ${\vec{t}}_{cam}^{\;tag\;T}$ from Equation (9) gives the distance between the follower and leader robots in the x and y directions expressed in the global coordinate frame. To calculate the angle between the leader and follower, the modification of the pitch angle $\theta_{R}$ of the ArUco marker is conducted, which is explained in Figure 6. The pose difference between the leader and follower robot is ($z_{marker},x_{marker},\overset{´}{\theta_{R}}$).

#### 3.3.2. Case 2—When Two Markers Detected

The proposed solution by the authors is the translation and rotation of markers to the center of the leader robots. Theoretically, both the detected markers should give the same readings, but due to manufacturing and pasting errors, the markers give slightly different readings of the center of the leader robot. The solution to the problem raised is to take the average readings of markers depending on the pixel areas captured in the image frame.

The pixel area $a_{i}$ of the i’th marker is calculated by the Shoelace formula [28] as:

(10) $$a=\frac{1}{2}\left|x_{1}y_{2}+x_{2}y_{3}+x_{3}y_{4}+x_{4}y_{1}-x_{1}y_{4}-x_{2}y_{1}-x_{3}y_{2}-x_{4}y_{3}\right|\;,$$

**Figure 7:**
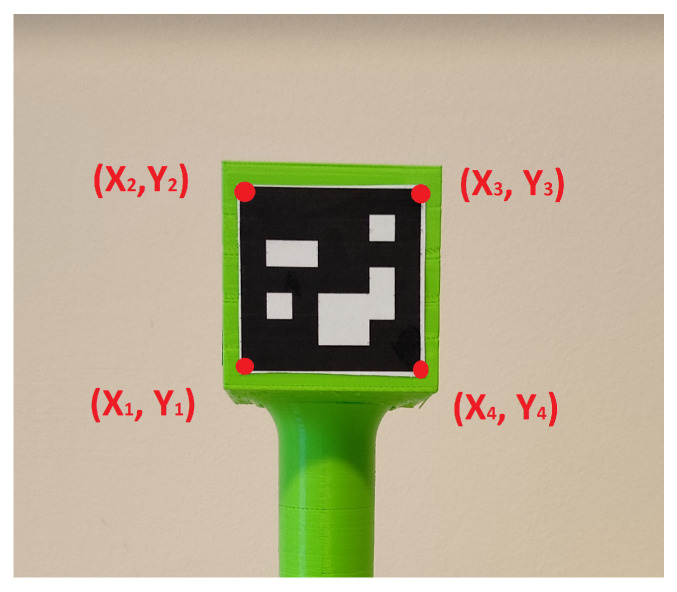
Representation of ArUco marker corner points.

The Shoelace formula is a mathematical algorithm to find out the area after cross multiplication of corresponding coordinates of vertices. Figure 7 explains the corner points of the ArUco markers. The pixels in the x coordinates are $\left(x_{1},\ldots ,x_{4}\right)$ and in the y coordinates are $\left(y_{1},\ldots ,y_{4}\right)$.

The total area A is calculated as follows:

(11) $$A=\sum_{i=1}^{N_{m}}a_{i}\;,$$

where $N_{m}$ is the total number of markers detected. At most, the follower robot may detect only two markers, so $N_{m}=2$.

Both the detected markers have sets of translation ${\vec{t}}_{vec\;i}$ and rotational $R_{TC\;i}$ vectors. The new rotation matrices $\overset{´}{R_{TC\;i}}$ are calculated as explained in Equation (6). Both of the detected markers come into the same XY planes after multiplying with rotation matrices. To obtain the center of the leader robot, translation distances should be added to translation ${\vec{t}}_{vec\;i}$ vectors. The distance of 0.03 m is added to the Z-axis. As the cuboidal side is 0.06 m, 0.06 m is added or subtracted according to the set of markers detected. When ArUco IDs (ID1 and ID2) or (ID2 and ID3) are detected, the translation distances are added in the following ways:

(12) $${\vec{\overset{´}{t}}}_{vec\;1}={\vec{t}}_{vec\;1}+{\left[\begin{array}{ccc} d_{x1} & d_{y1} & d_{z1} \end{array}]\right.}^{T},$$

where $d_{x1}=0$, $d_{y1}=0$ and $d_{z1}=0.03$.

(13) $${\vec{\overset{´}{t}}}_{vec\;2}={\vec{t}}_{vec\;2}+{\left[\begin{array}{ccc} d_{x2} & d_{y2} & d_{z2} \end{array}]\right.}^{T},$$

where $d_{x2}=0.06$, $d_{y2}=0$ and $d_{z2}=0.03$.

And when ArUco IDs (ID3 and ID4) or (ID4 and ID1) are detected:

(14) $${\vec{\overset{´}{t}}}_{vec\;1}={\vec{t}}_{vec\;1}+{\left[\begin{array}{ccc} d_{x1} & d_{y1} & d_{z1} \end{array}]\right.}^{T},$$

where $d_{x1}=0$, $d_{y1}=0$ and $d_{z1}=0.03$.

(15) $${\vec{\overset{´}{t}}}_{vec\;2}={\vec{t}}_{vec\;2}+{\left[\begin{array}{ccc} d_{x2} & d_{y2} & d_{z2} \end{array}]\right.}^{T},$$

where $d_{x2}=-0.06$, $d_{y2}=0$ and $d_{z2}=0.03$.

$\overset{´}{R_{TC\;1}}$ and $\overset{´}{R_{TC\;2}}$, ${\vec{\overset{´}{t}}}_{vec\;1}$ and ${\vec{\overset{´}{t}}}_{vec\;2}$ are used to calculate the translation matrix tags on the camera.

(16) $${\vec{t}}_{cam\;1}^{\;tag}=-\overset{´}{R_{TC\;1}}\cdot {\vec{\overset{´}{t}}}_{vec\;1}\;,$$

(17) $${\vec{t}}_{cam\;2}^{\;tag}=-\overset{´}{R_{TC\;2}}\cdot {\vec{\overset{´}{t}}}_{vec\;2}\;,$$

The average of translation vector $\bar{{\vec{t}}_{cam}^{\;tag}}$ is calculated as follows:

(18) $$\bar{{\vec{t}}_{cam}^{\;tag}}=\frac{1}{A}\sum_{i=1}^{N_{m}}a_{i}{\vec{t}}_{cam\;i}^{\;tag}\;.$$

In this case, the distances $z_{marker}$ and $x_{marker}$ are taken from the average matrix $\bar{{\vec{t}}_{cam}^{\;tag}}$ from Equation (18). The authors have considered the pitch angle $\theta_{R}$ from the marker having a bigger pixel area. After considering the pitch angle, the new angle $\overset{´}{\theta_{R}}$ is calculated as explained in Figure 6.

### 3.4. Pose Calculation of Leader Robot

**Figure 8:**
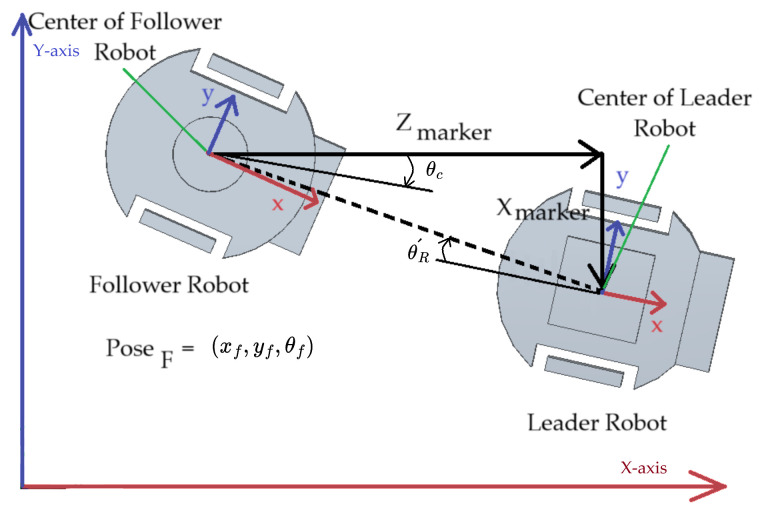
Pose representation of the leader and follower robots.

Let the poses of follower robot ($Pose_{F}$) be represented as $x_{2}$, $y_{2}$, and $\theta_{2}$. After ArUco marker detection, $z_{tag}$ and $x_{tag}$ give the distances in the x-axis and y-axis, respectively, between the center of the follower and leader robot. The angle between the camera and the ArUco marker is $\overset{´}{\theta_{R}}$ and the angle between the rotating platform and follower robot base is $\theta_{c}$. Both the angles $\theta_{c}$ and $\overset{´}{\theta_{R}}$ are added to obtain the resultant angle, as they are opposite in nature. Figure 8 represents the situation of models after pose estimation (Section 3.3) or the situation of robots assuming the estimation is perfect.

The pose of the leader robot ($Pose_{L}$) is written as:

(19) $$Pose_{L}^{⊤}=\left[\begin{array}{c} x_{1}\\ y_{1}\\ \theta_{1} \end{array}\right]=\left[\begin{array}{c} x_{2}+z_{marker}\\ y_{2}+x_{marker}\\ \theta_{2}+\overset{´}{\theta_{R}}+\theta_{c} \end{array}\right].$$

## 4. Extended Kalman Filter

**Figure 9:**
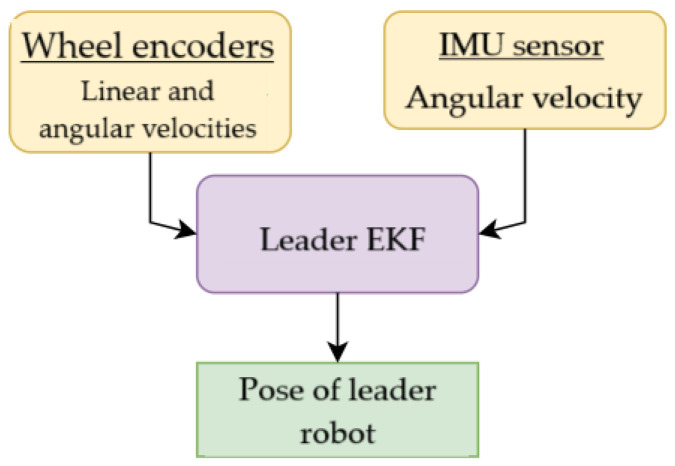
EKF of leader robot.

**Figure 10:**
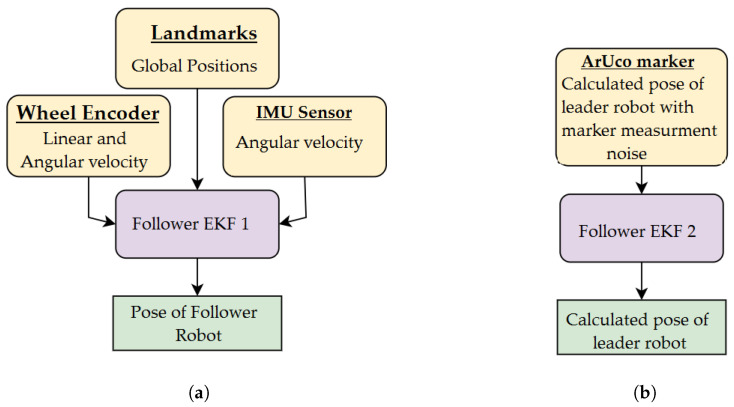
(**a**) EKF 1 of follower robot. (**b**) EKF 2 of follower robot.

A total of three Extended Kalman Filters (EKFs) are used in the robots—two EKFs in the follower robot and one EKF in the leader robot. The EKF of the leader robot (Figure 9) takes the input data from the wheel encoders and IMU sensor to generate the robot’s own poses in the global frame, whereas one of the follower EKFs (Figure 10a) has the same inputs but with landmarks in addition. The remaining EKF of the follower robot (Figure 10b) predicts the position of the leader robot after ArUco marker detection. The EKF model is taken from the paper [29]. The system state *X* of EKF is written as:

(20) $$X={\left[\begin{array}{ccccc} x_{i}\; & y_{i}\; & \theta_{i}\; & v_{i}\; & \omega_{i} \end{array}\right]}^{⊤}\;,$$

where i=1,2 for leader or follower robot. Variables $x_{i}$, $y_{i}$, and $\theta_{i}$ represent the position in the x-axis, y-axis, and the orientation of the leader or follower robot in the global frame, respectively. The linear and angular velocities are $v_{i}$ and $\omega_{i}$, respectively.

The discrete time model of the system $X\left(k\right)={\left[x_{i}\left(k\right)\;y_{i}\left(k\right)\;\theta_{i}\left(k\right)\;v_{i}\left(k\right)\;\omega_{i}\left(k\right)\right]}^{⊤}$ is written as follows:

(21) $$X\left(k\right)=\left[\begin{array}{c} x_{i}\left(k-1\right)+v_{i}\left(k\right)▵t\cos\theta_{i}\left(k-1\right)\\ y_{i}\left(k-1\right)+v_{i}\left(k\right)▵t\sin\theta_{i}\left(k-1\right)\\ \theta_{i}\left(k-1\right)+\omega_{i}\left(k\right)▵t\\ v_{i}\left(k-1\right)\\ \omega_{i}\left(k-1\right) \end{array}\right]\;,$$

The state model and measurement model of the filter are as follows:

(22) X(k)=f(X(k−1))+w(k−1),

(23) z(k)=h(X(k))+υ(k),

where the non-linear system is represented as *f*, measurement model as *h*, the dynamic system Gaussian noise as w(k−1) (w(k)∼N(0,Q(k))) and the measurement Gaussian noise as υ(k) (υ(k)∼N(0,R(k))). The covariance matrices are Q(k) and R(k). To make the system linearized, a Jacobian matrix of *f* is calculated as:

(24) $$F\left(k\right)=\frac{\partial f(X)}{\partial X}{|}_{{\hat{X}}_{k}^{-}}$$

(25) $$F\left(k\right)=\left[\begin{array}{ccccc} 1 & 0 & -v_{i}\left(k\right)▵t\sin\theta_{i}\left(k\right) & ▵t\cos\theta_{i}\left(k\right) & 0\\ 0 & 1 & v_{i}\left(k\right)▵t\cos\theta_{i}\left(k\right) & ▵t\sin\theta_{i}\left(k\right) & 0\\ 0 & 0 & 1 & 0 & ▵t\\ 0 & 0 & 0 & 1 & 0\\ 0 & 0 & 0 & 0 & 1 \end{array}\right]\;,$$

where ▵t is the time difference between the two consecutive samples. The prediction Equations are as follows:

(26) $$\hat{X}{\left(k\right)}^{-}=f\left(\hat{X}{\left(k-1\right)}^{+}\right)\;,$$

(27) $$P{\left(k\right)}^{-}=F\left(k\right)P\left(k-1\right)F{\left(k\right)}^{T}+Q\left(k\right)\;,$$

The update Equations are as follows:

(28) $$K\left(k\right)=P{\left(k\right)}^{-}H{\left(k\right)}^{T}{\left(H\left(k\right)P{\left(k\right)}^{-}H^{T}+R\left(k\right)\right)}^{-1}\;,$$

(29) $$\hat{X}{\left(k\right)}^{+}=\hat{X}{\left(k\right)}^{-}+K\left(k\right)\left(z\left(k\right)-h\left(\hat{X}{\left(k\right)}^{-}\right)\right)\;,$$

(30) $$P{\left(k\right)}^{+}=\left(I-K\left(k\right)H\left(k\right)\right)P{\left(k\right)}^{-}\;.$$

where the Kalman gain is represented as K(k), $\hat{X}{\left(k\right)}^{-},P{\left(k\right)}^{-},\hat{X}{\left(k\right)}^{+}$ and $P{\left(k\right)}^{+}$ are priori and posteriori state estimates.

### 4.1. Wheel Encoder Data of the EKF

Both the leader and follower robots have motors with encoders. As a result, the wheel angular velocities of the left and right wheel in radians per second are calculated as:

(31) $$w_{li}=\left(\phi_{c}l_{i}-\phi_{p}l_{i}\right)/▵t_{\phi }l\cdot \pi /180\;,$$

(32) $$w_{ri}=\left(\phi_{c}r_{i}-\phi_{p}r_{i}\right)/▵t_{\phi }r\cdot \pi /180\;,$$

where $w_{li}$ and $w_{ri}$ are the angular velocities (rad/s) of the left and right wheels of *i*-th robot. $\phi_{c}l_{i}$ and $\phi_{p}l_{i}$ are current encoder ticks and previous saved encoder ticks, respectively, for the left wheels, whereas $\phi_{c}r_{i}$ and $\phi_{p}r_{i}$ are for the right wheels. $▵t_{\phi }l$ and $▵t_{\phi }r$ are the time between the two consecutive readings of the left and right wheel readings.

The linear velocities (in m/s) of wheels are calculated by multiplying the wheel radius $R_{wheel\;i}$ in Equations (31) and (32) as:

(33) $$V_{li}=w_{li}\cdot R_{wheel\;i}\;,$$

(34) $$V_{ri}=w_{ri}\cdot R_{wheel\;i}\;,$$

Equations (33) and (34) are used to calculate the linear and angular velocities of robots as:

(35) $$v_{wheel\;i}=\left(V_{ri}+V_{li}\right)/2\;,$$

(36) $$\omega_{wheel\;i}=\left(V_{ri}-V_{li}\right)/L_{i}\;,$$

where $L_{i}$ is the length of the wheel separation of robots in meters. $v_{wheel\;i}$ and $\omega_{wheel\;i}$ are linear and angular velocities of robots calculated from the wheel encoder readings. The calculated linear and angular velocities of robots are used in EKF to calculate the pose of the robot in the global reference frame.

The measurement matrix $z_{encoder}\left(k\right)$ for EKF is as follows:

(37) $$z_{encoder}\left(k\right)=\left[\begin{array}{cc} v_{wheel\;i} & \omega_{wheel\;i} \end{array}\right]\;,$$

The $H_{encoder}\left(k\right)$ matrix is as follows:

(38) $$H_{encoder}\left(k\right)=\left[\begin{array}{ccccc} 0 & 0 & 0 & 1 & 0\\ 0 & 0 & 0 & 0 & 1 \end{array}\right]\;.$$

### 4.2. IMU Data to the EKF

The gyroscope data are only used from the IMU sensor in both robots. The measurement matrix $z_{gyro}\left(k\right)$ for IMU data is as follows:

(39) $$z_{gyro}\left(k\right)=\left[\begin{array}{c} \omega_{gyroscope\;i} \end{array}\right]\;,$$

The $H_{gyro}\left(k\right)$ matrix is as follows:

(40) $$H_{gyro}\left(k\right)=\left[\begin{array}{ccccc} 0 & 0 & 0 & 0 & 1 \end{array}\right]\;.$$

### 4.3. Landmark Data to the EKF

ArUco markers give the global x and y positions of robots, so $z_{landmark}\left(k\right)$ is as follows:

(41) $$z_{landmark}\left(k\right)=\left[\begin{array}{cc} x_{i} & y_{i} \end{array}\right]\;,$$

The $H_{landmark}\left(k\right)$ matrix is as follows:

(42) $$H_{landmark}\left(k\right)=\left[\begin{array}{ccccc} 1 & 0 & 0 & 0 & 0\\ 0 & 1 & 0 & 0 & 0 \end{array}\right]\;.$$

### 4.4. Pose Prediction of Leader Robot

The follower robot has another EKF which predicts the position of the leader robot. The measurement matrix $z_{leader}\left(k\right)$ for leader pose prediction is as follows:

(43) $$z_{leader}\left(k\right)=\left[\begin{array}{ccc} x_{1} & y_{1} & \theta_{1} \end{array}\right]\;,$$

where the pose of the leader robot ($x_{1},y_{1},\theta_{1}$) is taken from Equation (19). The $H_{leader}\left(k\right)$ matrix is as follows:

(44) $$H_{leader}\left(k\right)=\left[\begin{array}{ccccc} 1 & 0 & 0 & 0 & 0\\ 0 & 1 & 0 & 0 & 0\\ 0 & 0 & 1 & 0 & 0 \end{array}\right]\;.$$

## 5. Control Algorithm

### 5.1. Control for Rotating Platform

The movement of the rotating platform on the follower robot is independent of its robot body. A proportional–integral–derivative (PID) controller is used to control the movement of the rotating platform. The main aim of the controller is to minimize the distance between the center of the camera and the ArUco marker as much as possible. The distance between the ArUco marker center and the camera center ($x_{marker}$) is either taken from Equation (8) or (18) depending on the ArUco marker.

(45) $$u_{PID}\left(t\right)=K_{P}e\left(t\right)+K_{i}\int e(t)dt+K_{d}\frac{de}{dt}\;,$$

where $u_{PID}\left(t\right)$ is the control signal generated for a time *t*. $K_{P}$, $K_{i}$ and $K_{d}$ are the proportional gain, integral gain, and derivative gain parameters, de is the change in error, dt is the change in the time, and e(t) is expressed as follows:

(46) e(t)=SP−PV(t).

where SP is the setpoint which is set to be zero as the camera should point between the center of the ArUco marker. PV(t) is the process variable which was equal to the current angle between the center of the camera and the ArUco marker ($x_{marker}$).

### 5.2. Control Leader and Follower Robots

The kinematic model of the mobile robot $R_{i}$ (i=1,2) is written as:

(47) $$\left[\begin{array}{c} \dot{x_{i}}\\ \dot{y_{i}}\\ \dot{\theta_{i}} \end{array}\right]=\left[\begin{array}{cc} \cos\theta_{i} & 0\\ \sin\theta_{i} & 0\\ 0 & 1 \end{array}\right]\left[\begin{array}{c} v_{i}\\ \omega_{i} \end{array}\right]\;\;,$$

where i=1,2 for the leader and follower robots, respectively. The robot pose is represented by vector ${\left[x_{i}\;\;y_{i}\;\;\theta_{i}\right]}^{⊤}$. $x_{i}$, $y_{i}$, and $\theta_{i}$ are the variables representing the pose of the robot in the global reference frame. The control vector is ${\left[\begin{array}{cc} v_{i} & \omega_{i} \end{array}\right]}^{⊤}$, where $v_{i}$ and $\omega_{i}$ are linear and angular velocity controls of the robot, respectively. The aim of the leader is to mimic the pose ${\left[x_{0}\;\;y_{0}\;\;\theta_{0}\right]}^{⊤}$ of the virtual leader:

(48) $$\begin{array}{cc} x_{1d} & =x_{0}\\ y_{1d} & =y_{0}\\ \theta_{1d} & =\theta_{0}. \end{array}$$

The desired velocity vector is ${\left[v_{0}\;\;\omega_{0}\right]}^{⊤}$, where $v_{0}$ is the linear velocity and $\omega_{0}$ is the angular velocity. With some constant displacement ${\left[d_{2x}\;\;d_{2y}\right]}^{⊤}$, the follower has to mimic the motion of the leader on a mobile platform:

(49) $$\begin{array}{c} x_{2d}=x_{1}+d_{2x}\\ y_{2d}=y_{1}+d_{2y}\;, \end{array}$$

and with the same orientation:

(50) $$\begin{array}{c} \theta_{2d}=\theta_{1}\;, \end{array}$$

which brings the following quantities to zero:

(51) $$\begin{array}{ll} p_{ix} & =x_{id}-x_{i}\\ p_{iy} & =y_{id}-y_{i}\\ p_{i\theta } & =\theta_{id}-\theta_{i}. \end{array}$$

The system errors with respect to the robot’s fixed frame are as follows:

(52) $$\left[\begin{array}{c} e_{ix}\\ e_{iy}\\ e_{i\theta } \end{array}\right]=\left[\begin{array}{ccc} \cos(\theta_{i}) & \sin(\theta_{i}) & 0\\ -\sin(\theta_{i}) & \cos(\theta_{i}) & 0\\ 0 & 0 & 1 \end{array}\right]\left[\begin{array}{c} p_{ix}\\ p_{iy}\\ p_{i\theta } \end{array}\right].$$

Due to its simplicity and its effectiveness, the trajectory tracking algorithm is taken from [30]. In the original version [30], the algorithm was used for trajectory tracking by a single robot. The same paper presents stability proof that remains valid for the leader without changes and for the follower under the assumption that the leader’s motion is a trajectory generator for the follower after adding a fixed displacement ${\left[d_{2x}\;\;d_{2y}\right]}^{⊤}$. The control for the *i*-th robot is written as:

(53) $$\begin{array}{c} v_{i}=v_{i-1}\cos e_{i\theta }+k_{1}e_{ix}\\ \omega_{i}=\omega_{i-1}+k_{2}\mathrm{sgn}\left(v_{i-1}\right)e_{iy}+k_{3}e_{i\theta }, \end{array}$$

where $k_{1}$, $k_{2}$ and $k_{3}$ are constant parameters greater then zero, and the function sgn(•) is defined as follows:

$$\mathrm{sgn}\left(\xi \right)=\left\{\begin{array}{ccc} -1 & \mathrm{for} & \xi <0\\ 0 & \mathrm{for} & \xi =0\\ 1 & \mathrm{for} & \xi >0 \end{array}\right..$$

A detailed analysis of the properties of the control is presented in paper [30].

## 6. Methodology

The experiments with real robots are performed in an environment without obstacles. A PC with Intel i7 5th generation with 16 GB RAM and Ubuntu 20.04 as the operating system is used. ROS Noetic is installed on the PC and both the two robots. A NUC single-board computer is used in both robots as the hardware. PC and both the robots are connected to the same WiFi network. PC is selected as the ROS master and both robots are connected as the slave nodes. The task for the formation of two robots is to follow a sinusoidal trajectory. Two experiments were performed by the authors.

In experiment 1 (Section 7.1), the follower robot has to follow the leader as it will move along sinusoidal path. The behavior tree and landmarks are not used in the experiments, the experiment is performed to validate the algorithm presented by the authors in which the follower robot will detect the markers on the leader robot and calculate its global pose. The rotation platform will continuously observe the markers on the leader robot with the PID controller described in the paper. All the data generated in the experiments were saved in the text (txt) files.

In experiment 2 (Section 7.2), landmarks are placed in the environment. The behavior Tree will switch the movement of the rotating platform towards landmarks placed on the wall or to towards the leader robot.

## 7. Experiment Results

The control gains of the controller (53) between the virtual robot and leader robot are $k_{1}=0.15$, $k_{2}=2$ and $k_{3}=2$. The control gains of the controller (53) between the leader robot and follower robot are $k_{1}=0.25$, $k_{2}=1.5$ and $k_{3}=1.5$. Their values were tuned manually. The $d_{2x}=0.5$ and $d_{2y}=0$ in Equation (49).

### 7.1. Experiment 1

**Figure 11:**
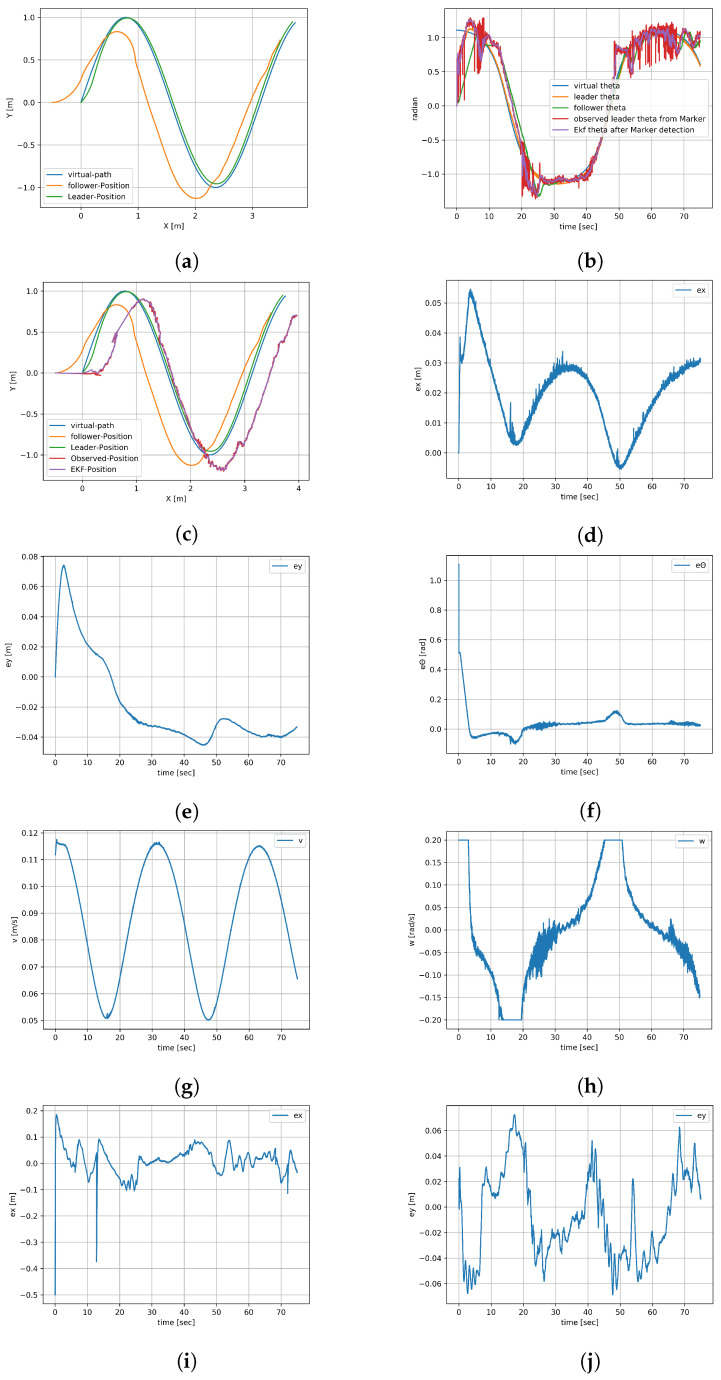
Experiment result 1 (**a**). (**a**) (x,y) plot of all robots. (**b**) Theta of all the robots. (**c**) (x,y) plot of all robots with EKF. (**d**) Error in x-axis for leader robot. (**e**) Error in y-axis for leader robot. (**f**) Error of leader’s orientation. (**g**) Linear velocity of leader robot. (**h**) Angular velocity of leader robot. (**i**) Error in x-axis for follower robot. (**j**) Error in y-axis for follower robot.

**Figure 12:**
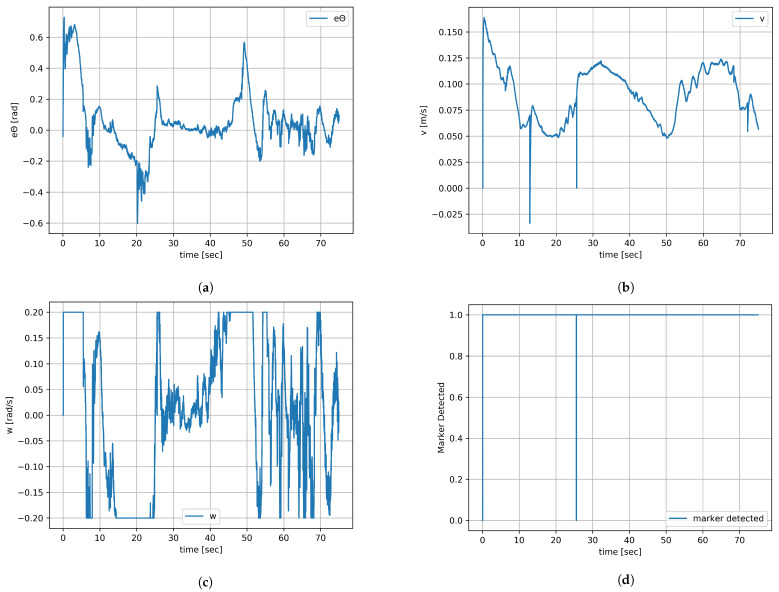
Experiment result 1 (**b**). (**a**) Error of follower’s orientation. (**b**) Linear velocity of follower robot. (**c**) Angular velocity of follower robot. (**d**) Marker detection information.

Experiment 1’s duration was 75 s. Figure 11a shows the (x,y) plots of the virtual leader, the leader robot, and the follower robot. The pose theta of all the robots is represented in Figure 11b, which also represents the EKF output of the theta after filtering. The noise is visible on the plot of the orientation from EKF after marker detection is caused by the inaccuracy of mounting and 3D printing of the cuboidal stand and the limited precision of sticking the ArUco markers onto it. The noise is particularly noticeable when two ArUco markers, placed on two perpendicular walls of the stand are observed at the same time. As the leader robot tries to mimic the pose of the virtual robot, the leader–follower controller tries to minimize the desired displacement which is a design parameter between the robots. The errors of the leader robot in the *x*-axis, *y*-axis, and orientation are shown in Figure 11d, Figure 11e and Figure 11f, respectively. Their values do not exceed 7 cm. The actual linear and angular velocities of the leader robot are shown in Figure 11g and Figure 11h, respectively. For short periods, angular velocity reaches the platform limit set at 0.2 rad/s. The follower robot detects the ArUco markers on the leader robot and tries to mimic the pose of the leader robot, and the errors generated by the leader–follower controller are saved and plotted. The errors of the follower robot in the *x*-axis, *y*-axis, and orientation are shown in Figure 11i,j and Figure 12a, respectively. The actual linear and angular velocities of the follower robot are shown in Figure 12b and Figure 12c, respectively. The noise associated with ArUco marker detection and, consequently, the result of relative position computation translates into more variable waveforms than in the case of the leader robot and also more frequent reaching of platform velocity limits. The follower robot that observed the pose of the leader robot through ArUco marker detection after EKF is shown in Figure 11c. The information from the follower robot, whether it detected the ArUco marker or not, is shown in Figure 12d; 1 means the marker is detected and 0 means the marker is not detected in the figure. As can be seen, the ArUco marker was not visible for only a short while.

### 7.2. Experiment 2

**Figure 13:**
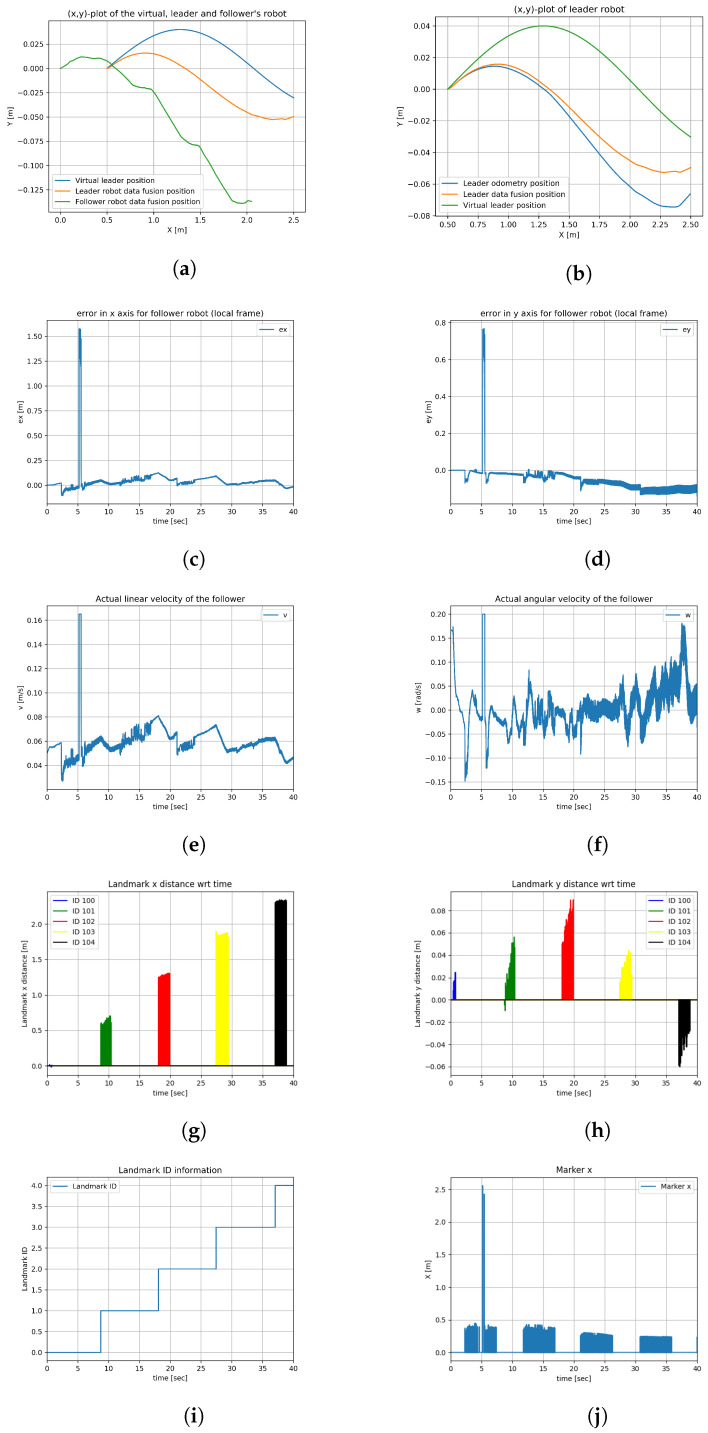
Experiment result 2 (**a**). (**a**) (x,y) plot of all robots. (**b**) (x,y) plot of leader robot. (**c**) Error in x-axis for follower robot. (**d**) Error in y-axis for follower robot. (**e**) Linear velocity of follower robot. (**f**) Angular velocity of follower robot. (**g**) Landmark x-distance. (**h**) Landmark y-distance. (**i**) Landmark id information. (**j**) Marker x distance.

**Figure 14:**
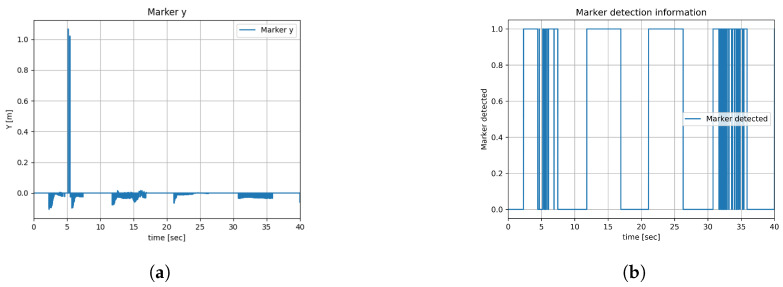
Experiment result 2 (**b**). (**a**) Marker y distance. (**b**) Marker detection information.

Experiment 2’s duration was 40 s. Figure 13a shows the (x,y) plots of the virtual leader, the leader robot, and the follower robot. The path of the follower robot is shown in green in Figure 13a, which shows disturbances in the sinusoidal trajectory; for 3 s, the follower robot watched the landmarks on the walls, and for 5 s the follower robot watched the leader robot. Figure 13b shows the (x,y) plots of the virtual leader, odometry, and data fusion positions of the leader robot. A slight drift from the odometry data is removed after data fusion. The errors generated from the leader–follower controller algorithm are saved and plotted. The errors of the follower robot while following the leader robot in the *x*-axis and *y*-axis are shown in Figure 13c and Figure 13d, respectively. The actual linear and angular velocities of the follower robot are shown in Figure 13e and Figure 13f, respectively. Figure 13g,h show the distance of the follower robot in the global coordinate frame when the camera on the rotating platform observes the landmarks. Figure 13i gives the information of the landmark ID detected. Figure 13j and Figure 14a show the distance between the follower robot and the leader robot when the camera on the rotation platform of the follower robot watches the leader robot. The information from the follower robot, whether it detected the ArUco marker or not, is shown in Figure 14b; 1 means the marker is detected and 0 means the marker is not detected in the graph.

## 8. Conclusions

A leader–follower approach based on extended onboard camera perception is shown in this paper with the integration of a behavior tree and EKF-based sensor data fusion. The behavior tree was on the top-level controller, which controlled the robots in the workspace. When the follower robot is ready in the environment, the behavior tree sends the signals to the leader robot to mimic the virtual robot. The one-degree-of-freedom rotating platform on the follower robot is also controlled by the behavior tree. For 5 s, the rotating platform watched the leader robot, and for 3 s it watched the landmark. A low-level PID control for the ArUco marker has followed the markers on the leader robot. The leader robot followed the virtual leader robot and as a result, mimicked the sine curve of the virtual leader robot.

The authors have performed two experiments. In one experiment, the landmarks and behavior tree are not involved, so the rotation platform of the follower robot observes the marker on the leader continuously. The result of the experiment shows that the leader robot followed the virtual robot with a diverging error in the *x* and *y* axes of less than 4 cm. The follower robot followed the leader robot with a diverging error in the *x* and *y* axes of less than 4 cm. The EKF has filtered out the data, which is clearly seen in Figure 11b,c. In the second experiment, all the algorithms proposed in the paper are involved. The behavior tree controlled the switching of the rotating platform from landmarks to markers and vice versa. The global position errors for the *x* and *y* axes for the follower robot diverge towards approximately zero. Data fusion of leader and follower robots with the use of EKF is achieved. The fluctuation in the follower robot is seen in the results due to the landmark data. In future research, authors of this paper will work to minimize the fluctuations due to the landmark addition. The follower robot should achieve exactly the same trajectory as that of the leader without disturbances, which will also be another challenging task for the future.

## Data Availability

Not applicable.
